# The Adaptor Protein Lurap1 Is Required for Cell Cohesion during Epiboly Movement in Zebrafish

**DOI:** 10.3390/biology10121337

**Published:** 2021-12-16

**Authors:** Ji-Tong Li, Xiao-Ning Cheng, Chong Zhang, De-Li Shi, Ming Shao

**Affiliations:** 1 School of Life Sciences, Shandong University, Qingdao 266237, China; lijt@mail.sdu.edu.cn (J.-T.L.); zhangchong@mail.sdu.edu.cn (C.Z.); 2 Laboratory of Zebrafish Model for Development and Disease, Affiliated Hospital of Guangdong Medical University, Zhanjiang 524001, China; chengxiaoning@mail.sdu.edu.cn; 3 Laboratory of Developmental Biology, CNRS-UMR7622, Institut de Biologie Paris-Seine, Sorbonne University, 75005 Paris, France

**Keywords:** epiboly, gastrulation, Lurap1, cell adhesion, cytoskeleton, F-actin, zebrafish

## Abstract

**Simple Summary:**

Cell adhesion and active cell shape changes play an important role in morphogenetic movements during embryonic development. Zebrafish is an attractive model for the study of cellular and molecular mechanisms underlying these processes. Epiboly is a conserved gastrulation cell movement, which describes the thinning and spreading of an external sheet of cells to cover other groups of cells in the embryo. It involves differential cellular adhesive properties and dynamic cytoskeletal organization across the embryo, but how these are regulated remains elusive. We found that the adaptor protein Lurap1, which interacts with other proteins required for cell migration, plays a role in cell adhesion during epiboly. In zebrafish mutants with loss of Lurap1 function, there is a reduced cellular cohesion in the epithelial blastoderm cells and a delayed epiboly movement. Our observations suggest that Lurap1 is implicated in the regulation of cellular behavior changes for coordinated morphogenetic movements in vertebrate embryos.

**Abstract:**

Cell adhesion and polarized cellular behaviors play critical roles in a wide variety of morphogenetic events. In the zebrafish embryo, epiboly represents an important process of epithelial morphogenesis that involves differential cell adhesion and dynamic cell shape changes for coordinated movements of different cell populations, but the underlying mechanism remains poorly understood. The adaptor protein Lurap1 functions to link myotonic dystrophy kinase-related Rac/Cdc42-binding kinase with MYO18A for actomyosin retrograde flow in cell migration. We previously reported that it interacts with Dishevelled in convergence and extension movements during gastrulation. Here, we show that it regulates blastoderm cell adhesion and radial cell intercalation during epiboly. In zebrafish mutant embryos with loss of both maternal and zygotic Lurap1 function, deep cell multilayer of the blastoderm exhibit delayed epiboly with respect to the superficial layer. Time-lapse imaging reveals that these deep cells undergo unstable intercalation, which impedes their expansion over the yolk cell. Cell sorting and adhesion assays indicate reduced cellular cohesion of the blastoderm. These defects are correlated with disrupted cytoskeletal organization in the cortex of blastoderm cells. Thus, the present results extend our previous works by demonstrating that Lurap1 is required for cell adhesion and cell behavior changes to coordinate cell movements during epithelial morphogenesis. They provide insights for a further understanding of the regulation of cytoskeletal organization during gastrulation cell movements.

## 1. Introduction

Morphogenetic movements of gastrulation play a critical role in actively moving various cell populations of the early embryo to their future positions, thereby setting up the three germ layers and elongating the anteroposterior axis. These fundamental developmental processes are evolutionarily conserved and mainly involve epiboly, invagination, involution, convergence and extension, and directed collective migration [1]. In the zebrafish embryo, epiboly is the earliest morphogenetic movement that starts at the end of the blastula period when the blastoderm consists of approximately 4000 cells located on the large yolk cell [2]. During the early stages of this movement, the large yolk cell elevates toward the animal pole by a process called doming. This produces a blastoderm of uniform thickness that consists of a flattened enveloping monolayer (EVL) and an underlying deep cell multilayer (DEL) of at least four cells thick [2]. Epiboly continues during the entire gastrulation period. Along with other cell movements, both EVL and DEL cells undergo active cell shape changes and rearrangements to coordinately spread toward the vegetal pole and cover the yolk cell by the end of gastrulation [3,4].

Radial intercalation of DEL cells plays an important role to expand the blastoderm surface during epiboly. It was thought that this is caused by the outward movement of more deep cells in the DEL to integrate into more superficial cells [2], but there is also evidence indicating bidirectional movements of DEL cells during radial intercalation [5,6]. Differential cell–cell adhesion in the blastoderm mediated by cell adhesion molecules is required for radial intercalation [7]. In *E-cadherin* mutant (*half-baked*) embryos, the DEL, but not the EVL, fails to complete epiboly, and convergence and extension are also delayed [8,9,10]. In these mutant embryos, although more deep cells of the DEL can intercalate with more superficial cells, they eventually move back, without expanding the blastoderm surface. Thus, E-cadherin plays a role in cell intercalation through regulation of cell shape changes, which correlates with its increasing expression levels from more deep cells toward more superficial cells within the DEL [8]. Moreover, in the absence of E-cadherin, the DEL shows abnormal adhesion with the EVL, which impairs the coordinated movements of these cell layers during epiboly. This suggests that the adhesive interaction between the DEL and the EVL is important for blastoderm expansion. Consistently, the basal surface of the EVL at the animal pole region displays dynamic actin-based formation of filopodia that may promote DEL rearrangements [11], indicating an active contribution of the cytoskeleton to cellular behavior changes. Indeed, dynamic changes in the cytoskeletal organization occur across the embryo and throughout epiboly movement [12]. In the zebrafish blastula and gastrula, cortical F-actin belt is organized in blastoderm cells, and F-actin bundles are also present in the vegetal cortex of the yolk cell until before the end of epiboly. Moreover, actomyosin contractile rings are elaborated at the blastoderm margin during the late stages of epiboly [13]. These actin cytoskeletons are involved in coordinating the vegetal movement of both EVL and DEL [14,15,16,17,18,19], but how their organization is regulated in the embryo remains elusive.

The adaptor protein Lurap1 (leucine repeat adaptor protein 1), previously called Lrap35a, displays two leucine-rich repeats at its N-terminal region and a PDZ-binding motif at the C-terminus. In cultured cells, it participates in the regulation of actomyosin retrograde flow and cell migration by forming a tripartite complex with myotonic dystrophy kinase-related Rac/Cdc42-binding kinase (MRCK) and the PDZ-containing unconventional MYO18A, through its leucine-rich repeats and PDZ-binding motif, respectively [20]. However, its implication in adhesion-mediated developmental processes is quite open for detailed investigation. We previously generated a zebrafish *lurap1* mutant line and found that mutant embryos displayed defective convergence and extension movements during gastrulation [21]. In the present study, detailed phenotypic analyses revealed that maternal-zygotic (MZ) *lurap1* (MZ*lurap1*) mutant embryos also exhibited epiboly delay in the DEL. Specifically, loss of Lurap1 function prevented stable radial cell intercalation in the DEL and reduced cell cohesion in the blastoderm. These abnormal cellular behaviors were associated with a disorganization of cortical F-actin belt, particularly in the DEL. Our results identify an essential role for Lurap1 in cellular behavior changes during epithelial morphogenesis.

## 2. Materials and Methods

### 2.1. Zebrafish

Zebrafish adults were maintained at 26–29 °C in standard housing systems (Haisheng, Shanghai, China) with a 14 h light and 10 h dark cycle. They were fed with brine shrimp twice daily. Mutant fish pairs from different batches that produced offspring with similar expressivity were used throughout this work. In each experiment, roughly equal numbers of wild-type and mutant embryos spawned at an interval of 10 min were cultured in a temperature-controlled incubator. Their synchronous development was controlled by examining the number of blastomeres at 8-cell to 32-cell stages. This project was approved by the Ethics Committee for Animal Research of Life Science of Shandong University (SYDWLL-2018–05).

### 2.2. Capped mRNA Synthesis and Microinjections

Expression constructs include pCS2-*lurap1*, pCS2-mRFP, pCS2-mGFP and pCS2-Kaede-GFP. Capped mRNAs were synthesized by in vitro transcription using SP6 RNA polymerase (Roche Diagnostics, Shanghai, China) in the presence of 500 µM of 5′-mGpppG-3′ cap analog (New England Biolabs, Beijing, China), 500 µM of rUTP, rATP and rCTP, and 100 µM of rGTP (Roche Diagnostics, Shanghai, China). Synthetic mRNA in 2–3 nL was injected at the 1-cell stage using a PLI-100A picoliter microinjector (Harvard Apparatus, Holliston, MA, USA). Whenever appropriate, an equal amount of GFP mRNA was used as a control for developmental delay due to microinjection and for successful microinjections.

### 2.3. UV-Mediated Photo-Conversion of Kaede GFP

Embryos at the 1-cell stage were injected with synthetic mRNA (100 pg) encoding Kaede GFP. At the sphere stage, UV-inducible green to red fluorescence conversion was performed at the animal pole region, with a DAPI filter using the smallest pinhole under the 20× objective of an upright microscope (Leica, LM2500, Mannheim, Germany). Live images were taken at 30 min intervals to follow the spreading of blastoderm cells. In all experiments, unlabeled embryos that were subjected to the same treatments were used to check the presence of autofluorescence. No differences were noticed between different treatments.

### 2.4. Cell Transplantation

Donor embryos were labeled with membrane GFP (mGFP) by injecting the corresponding mRNA (300 pg) at the 1-cell stage, and allowed to develop to the sphere stage. A small group of cells in the blastoderm were aspirated using the PLI-100A microinjector and transplanted to the animal pole region of unlabeled recipients at the same stage. Labeled cells were live-imaged at the shield stage.

### 2.5. Immunofluorescence

Embryos were fixed in 4% paraformaldehyde for 1 h at room temperature. They were incubated with rabbit polyclonal antibodies against ß-catenin (1/1000; Abcam, Shanghai, China), followed by fluorescein-conjugated secondary antibody. Labeled embryos were imaged using a confocal microscope (Zeiss, LSM700, Mannheim, Germany). To observe deep cells, the focal plane was set at 8–10 µm under the EVL at the blastoderm margin.

### 2.6. Analysis of EVL and DEL Epiboly

Stage-matched wild-type and MZ*lurap1* mutant embryos were stained with rhodamine-conjugated phalloidin and DAPI (Sigma-Aldrich, St Louis, MO, USA). Z-stack projections of confocal images were generated using the z-projection function. The extent of epiboly progression was determined by measuring the distance from the animal pole to the EVL or the DEL margin, with respect to the distance from the animal pole to the vegetal pole. Cortical F-actin organization in the animal pole region was also examined by phalloidin staining. Images were acquired using a confocal microscope (Zeiss, LSM700, Mannheim, Germany).

### 2.7. Live Time-Lapse Imaging

Wild-type and MZ*lurap1* mutant embryos at the 50% epiboly stage were mounted in a cavity microscope slide in 1% low-melting agarose. Cell movements in the outermost layer of the DEL located at the animal pole region were recorded at 20 s intervals for a period of 20 min using an upright microscope (Leica DM2500,Mannheim, Germany) and the LAS V4.3 software (Leica, Mannheim, Germany). Time-lapse movies were generated using ImageJ software (NIH Image). The experiment was repeated thrice, using six wild-type and MZ*lurap1* mutant embryos from different batches, respectively.

### 2.8. Cell Sorting and Adhesion Assays

The hanging drop assay was used to test the ability of cell aggregation [22]. Wild-type and MZ*lurap1* embryos at the 1-cell stage were first labeled using the cell lineage tracer fluorescein-lysine dextran (FLDx) or rhodamine-lysine dextran (RLDx). Blastoderm cells were then dissociated at the 30% epiboly stage in Ca^2+^/Mg^2+^-free Ringer’s solution and suspended in 0.66× Leibovitz L-15 medium (Invitrogen, Shanghai, China) supplemented with 10% fetal bovine serum and penicillin-streptomycin. Equal numbers of differentially labeled cells were mixed in a small drop (20 µL) of this medium and deposited onto a CELLVIEW tissue culture dish (Greiner bio-one, Shanghai, China). The dish was then gently inverted and cultured for 12 h at 25 °C. Aggregates were briefly fixed by 4% paraformaldehyde and imaged by z-stacked confocal microscopy (Zeiss, LSM700, Mannheim, Germany).

Cell–cell adhesion was determined by cluster formation on fibronectin-coated substrate. Wild-type and MZ*lurap1* embryos at the 1-cell stage were injected with RLDx, and groups of 30 embryos at the sphere stage were thoroughly dissociated into single cells in Ca^2+^/Mg^2+^-free Ringer’s solution. Following several washes in DMEM medium, cell pellets were resuspended in the same medium. Cell numbers were controlled using a cell counting chamber and adjusted to the same concentration (2 × 10^5^ cells/mL) for each condition. Approximately 2 × 10^4^ dissociated cells were seeded on a 96-well plate previously coated with 100 µg/mL fibronectin (Sigma-Aldrich, St Louis, MO, USA). Clusters formed by at least 4 cells were counted using random views taken at 3 h and 6 h, and the surface area that they occupied was determined using the ImageJ software (NIH Image, MD, USA).

### 2.9. In Situ Hybridization

Antisense probe for *tbxta* (*ntla*) was synthesized by SP6 RNA polymerase from linearized plasmid and labeled with digoxigenin-11-UTP (Roche Diagnostics, Shanghai, China).

### 2.10. Statistical Analyses

All data were collected from at least three independent experiments using different batches of embryos and statistically analyzed using unpaired Student’s *t* test.

## 3. Results

### 3.1. Loss of Maternal Lurap1 Delays Epiboly Movement

We previously showed that *lurap1* is a maternal gene; zygotic homozygous *lurap1* mutants developed normally like wild-type siblings, whereas MZ*lurap1* mutant embryos showed impaired convergence and extension movements, suggesting that maternal Lurap1 activity is required for these processes [21]. In this study, a detailed phenotypic analysis indicated that epiboly was also delayed in these embryos. To exclude the possibility that variations in the exact timing of fertilization may introduce fluctuations in the extent of epiboly progression, we monitored the number of blastomeres at 8- to 32-cell stages and followed closely epiboly movement between time-matched or stage-matched wild-type and MZ*lurap1* mutant embryos by following standard criteria [2].

**Figure 1:**
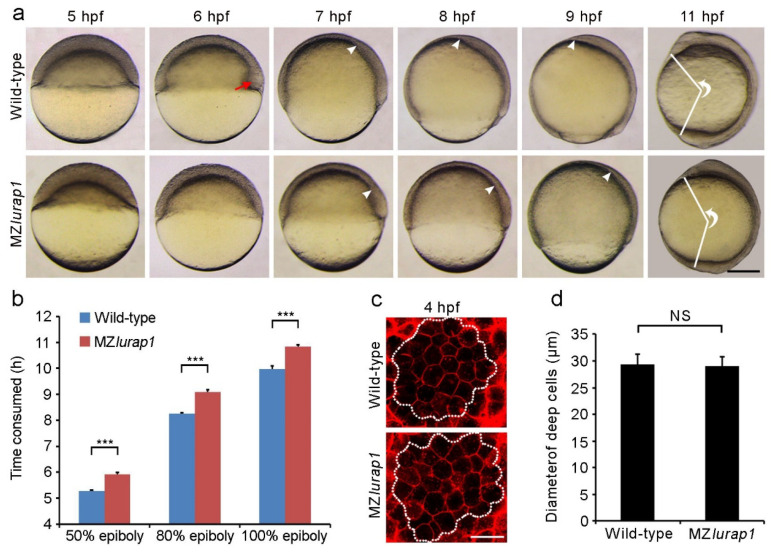
Delayed epiboly movement and reduced anteroposterior axis elongation in MZ*lurap1* mutant embryos. (**a**) Live images show epiboly progression in time-matched wild-type and MZ*lurap1* mutant embryos. Lateral view with animal pole or anterior region on the top. The red arrow indicates the embryonic shield; white arrowheads indicate the front of collective migration of prechordal mesendoderm. The extent of anteroposterior axis elongation is reflected by the angle formed between the most anterior end and the most posterior end, with vertex at the geometric centre of the embryo. Scale bar: 200 µm. (**b**) Comparison of the time interval to reach indicated epiboly stages between wild-type and MZ*lurap1* mutant embryos. Data were collected from three independent crosses, using a total of 150 embryos for each group. Bars represent the mean value ± s.d. (***, *p* < 0.001). (**c**) Examination of deep cell size at the animal pole region (dashed circles) in wild-type and MZ*lurap1* mutant embryos at 4 hpf. Scale bar: 50 µm. (**d**) Measurement of cell diameters by assuming that all cells are spherical. For each condition, data were obtained using 10 embryos from three independent crosses. Bars represent the mean value ± s.d. (NS, not significant).

The delayed epiboly phenotype in MZ*lurap1* mutant embryos could be observed before and during gastrulation in comparison with time-matched wild-type embryos (Figure 1a). At 5 hpf (hours post-fertilization), the blastoderm of MZ*lurap1* mutant embryos remained thicker when compared with time-matched wild-type embryos, indicating a slowed expansion over the yolk cell. At 6 hpf, wild-type embryos reached 50% epiboly and formed a thickened germ ring with the embryonic shield clearly visible on the future dorsal side (see Figure 1a, red arrowhead), whereas MZ*lurap1* mutant embryos did not show any dorsoventral difference. This suggests that both the convergence of lateral cells toward the dorsal region and the spreading of the blastoderm toward the vegetal pole are defective in the absence of Lurap1 function, which may result in a delayed internalization of hypoblast cells. Consistently, from 7 hpf to 9 hpf, the extent of involution and migration of prechordal mesendoderm cells toward the animal pole was obviously reduced in MZ*lurap1* mutant embryos. At 11 hpf, when somite formation became visible in wild-type embryos, MZ*lurap1* mutant embryos eventually completed gastrulation. However, they displayed a shortened anteroposterior axis, as reflected by the angle formed between the most anterior end and the most posterior end, with vertex at the geometric centre of the embryo. These observations demonstrate that in addition to its requirement for convergence and extension, the activity of maternal Lurap1 is also involved in epiboly movement.

**Figure 2:**
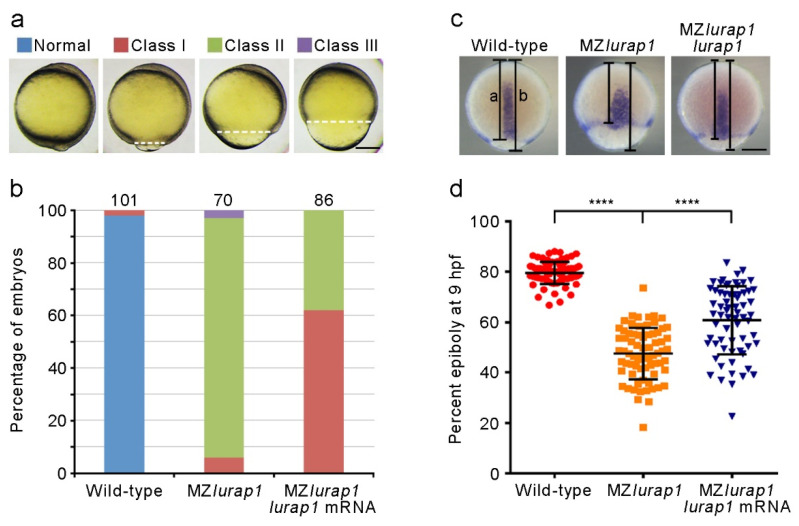
Rescue of epiboly delay in MZ*lurap1* mutant embryos by Lurap1. (**a**) Classification of the severity of epiboly delay at 10 hpf: normal embryos complete epiboly; class I embryos reach 90% epiboly; class II embryos show 75% to 90% epiboly; class III embryos fail to accomplish 75% epiboly. Broken lines indicate the leading edge of the blastoderm margin. Scale bar: 200 µm. (**b**) Graph shows the proportion of embryos with different degrees of epiboly delay. Numbers on the top of each stacked column represent total embryos scored from three independent experiments, and color keys are as indicated in (**a**). The results were analyzed independently in a blended manner. (**c**) Determination of the rescue efficiency by in situ hybridization analysis of *tbxta* to visualize the leading edge of the blastoderm margin at 9 hpf. Epiboly progression was calculated as the distance from the animal pole to the edge of the blastoderm margin (**a**) relative to the length of the animal-vegetal axis (**b**). Scale bar: 200 µm. (**d**) Scatter plot shows the rescue of epiboly progression in MZ*lurap1* mutant embryos by wild-type *lurap1* mRNA. Data are mean ± s.d. from three independent batches of embryos (****, *p* < 0.0001).

Analysis of the time interval to reach different epiboly stages revealed that MZ*lurap1* mutant embryos showed a delay of approximately 1 h in epiboly progression with respect to wild-type embryos when they developed at 28.5 °C (Figure 1b). This delay is significant given that the period from the initiation to the end of epiboly lasts 6 h at this temperature [2]. To exclude the possibility that the delayed epiboly progression in MZ*lurap1* mutant embryos may be caused by abnormal cell division during cleavage stages, we analyzed the size of membrane red fluorescence protein (mRFP)-labeled deep cells in the animal pole region of the blastoderm by confocal microscopy. This showed no difference in cell size between wild-type and MZ*lurap1* mutant embryos at 4 hpf, as determined by measurement of the cell diameter (Figure 1c,d), suggesting a specific effect on cell movements. In addition, the specificity of the epiboly phenotype was confirmed by rescue experiments. Injection of the full-length *lurap1* mRNA (100 pg) into MZ*lurap1* mutant embryos at the 1-cell stage was able to reduce the extent of epibloy delay, as determined by phenotypic assessment (Figure 2a,b) and by in situ hybridization analysis of the notochord marker *tbxta*, which helps to clearly visualize the leading edge of the blastoderm margin and the anterior extension of the chordamesoderm (Figure 2c,d). Thus, it can be concluded that loss of Lurap1 specifically causes defective cell movements during epiboly.

### 3.2. Delayed DEL Epiboly in MZlurap1 Mutants

**Figure 3:**
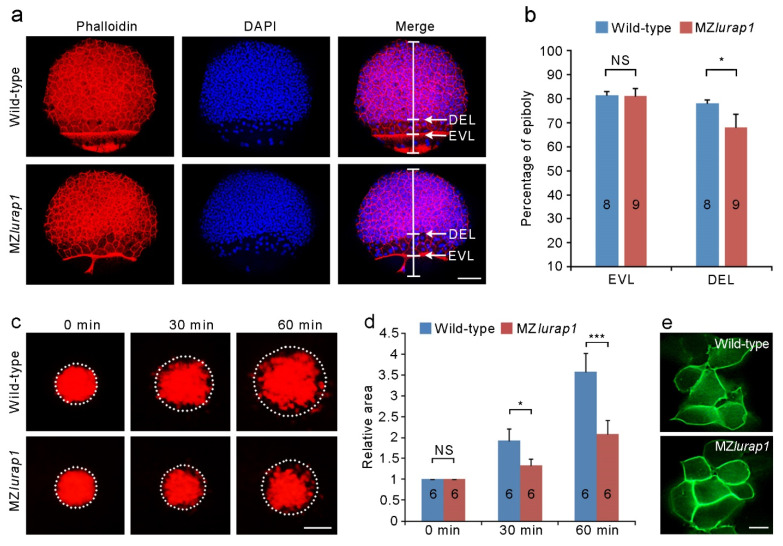
Delayed epibloy progression and reduced spreading capacity of DEL cells in MZ*lurap1* mutant embryos. (**a**) Phalloidin and DAPI staining of wild-type and MZ*lurap1* mutant embryos at 80% epiboly. Scale bar: 100 μm. (**b**) Statistical analysis of the extent of vegetal movements of the EVL and the DEL in wild-type and MZ*lurap1* mutant embryos. The percentage of epiboly was determined as the distance from the animal pole to the front of the EVL or the DEL margin with respect to the distance between the animal pole and the vegetal pole. Data were collected from 8 to 9 wild-type or MZ*lurap1* mutant embryos. Bar represents the mean value ± s.d. (NS, not significant; *, *p* < 0.05). (**c**) UV-induced green to red fluorescence conversion of Kaede GFP in the animal pole region at 4 hpf. Areas within dashed circles reflect the extent of DEL cell spreading at indicated times. Scale bar: 100 μm. (**d**) Statistical analysis of the extent of cell spreading. Data were collected from 6 embryos for each condition in three independent experiments, and the area initially occupied by labeled cells in wild-type embryos is normalized as 1. Bars represent the mean value ± s.d. (NS, not significant; *, *p* < 0.05; ***, *p* < 0.001). (**e**) Representative images of mGFP-labeled wild-type cells or MZ*lurap1* mutant cells transplanted to unlabeled wild-type recipients. Scale bar: 10 µm.

Radial cell intercalation in the DEL drives epiboly and allows the blastoderm to spread over the yolk cell. To see how the spreading behavior of the blastoderm was affected following loss of Lurap1 function, we first examined the progression of the EVL and the DEL margin by staining stage-matched wild-type and MZ*lurap1* mutant embryos with phalloidin and DAPI. When the leading edge of the EVL in both wild-type and MZ*lurap1* mutant embryos reached 80% epiboly, we found that the gap between the front of the DEL and the leading edge of the EVL in MZ*lurap1* mutant embryos was wider than that in wild-type embryos (Figure 3a), indicating that DEL epiboly was lagged further behind the EVL. Indeed, statistical analysis indicated a significant delayed DEL progression toward the vegetal pole in MZ*lurap1* mutant embryos (Figure 3b). Thus, loss of maternal Lurap1 mostly affects the spreading capacity of the DEL.

To directly examine this defective spreading behavior of the DEL, we labeled a small group of cells in the animal pole region through UV-inducible green to red fluorescence conversion of Kaede GFP at the sphere stage, and followed the expansion of the labeled area at different time points after UV conversion (Figure 3c). The focal plane was set under the EVL in order to observe underlying DEL cells. In wild-type embryos, active mixing occurred between labeled and unlabeled cells, such that the labeled area increased twice after 30 min and was nearly quadrupled after 60 min. By contrast, in MZ*lurap1* mutant embryos, labeled cells showed limited spreading after 30 min, and the area that they occupied increased only about twice after 60 min (Figure 3c,d). The defective spreading behaviors of MZ*lurap1* mutant cells was further confirmed by transplantation experiments. Wild-type and MZ*lurap1* mutant embryos were labeled with membrane green fluorescence protein (mGFP). At the shield stage, a small group of labeled cells were transplanted into the animal pole region of unlabeled wild-type embryos. When these mosaic embryos were examined at the shield stage, labeled wild-type cells showed loose contact and extended lamellipodia or filopodia, whereas MZ*lurap1* mutant cells remained in close contact and failed to form cellular protrusions (Figure 3e). These results suggest that loss of Lurap1 affects polarized cellular behaviors and cell shape changes in the DEL, which may lead to reduced intercalation during epiboly.

**Figure 4:**
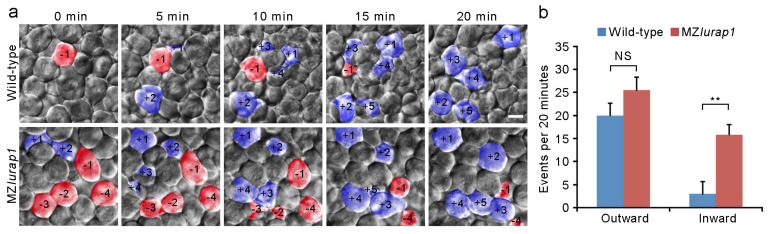
Defective radial intercalation of DEL cells in MZ*lurap1* mutant embryos. (**a**) Still frames from representative time-lapse imaging of DEL cell movements in the animal pole region of wild-type and MZ*lurap1* mutant embryos at 50% epiboly. The focal plane was set at the outermost layer of the DEL immediately underlying the EVL. Cells undergoing outward movements are fake color labeled blue, and are attributed with positive numbers, while cells undergoing inward movements are labeled red, and are attributed with negative numbers. Scale bar: 10 μm. (**b**) Statistical analysis compares inward and outward movements in the same focal plane during a period of 20 min. The results were analyzed independently in a blended manner. Data were collected from 5 embryos for each group in three independent experiments. Bars represent the mean value ± s.d. (NS, not significant; **, *p* < 0.01).

We next performed live time-lapse imaging to directly examine the behaviors of DEL cells in the animal pole region that exhibits most extensive radial cell intercalation [3]. When both wild-type and MZ*lurap1* mutant embryos reached 50% epiboly, cell-moving directions at the most superficial layer of the DEL, which is in direct contact with the basal surface of EVL cells, were monitored for a period of 20 min using six embryos from different batches for each condition. In wild-type embryos, the outward movement of more deep cells to the superficial layer largely outpaced the inward movement of cells present in the superficial layer (Figure 4a,b and Video S1 and Appendix A). By contrast, in MZ*lurap1* mutant embryos, balanced outward and inward movements could be observed in the superficial layer (Figure 4a,b and Video S2 and Appendix B). Thus, although there were no significant differences in the outward movement of more deep cells between wild-type and MZ*lurap1* mutant embryos, we found an increased inward movement of superficial cells in MZ*lurap1* mutant embryos (Figure 4b). This observation suggests that radial cell intercalation in the DEL is less efficient and becomes unstable in the absence of Lurap1 function, which may be responsible for its delayed epiboly with respect to the EVL.

### 3.3. Reduced Blastoderm Cell Cohesion in MZlurap1 Mutants

**Figure 5:**
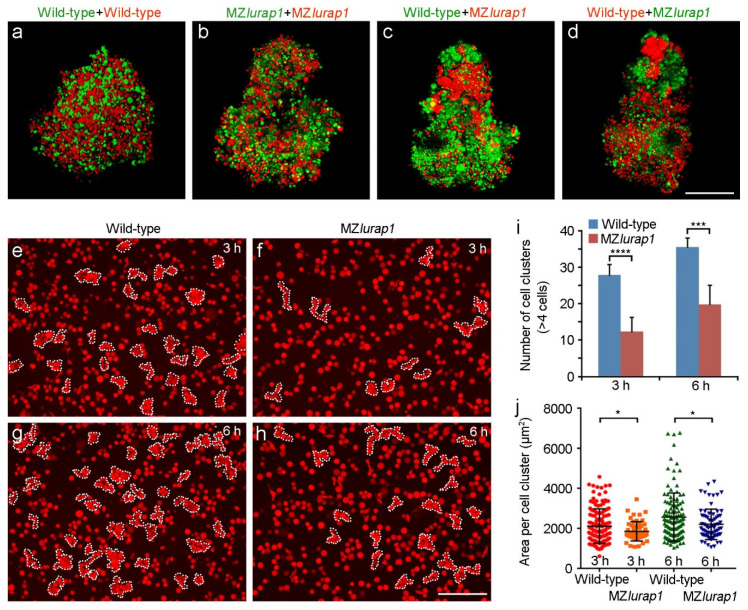
Reduced blastoderm cell cohesion in MZ*lurap1* mutant embryos. (**a**–**d**) Representative images from hang drop assay show differential affinity between wild-type and MZ*lurap1* mutant cells, as reflected by the preferential aggregation of cells with the same genotypes. Scale bar: 200 µm. (**e**–**h**) Representative images show cluster formation in wild-type and MZ*lurap1* mutant cells on fibronectin-coated substrates. At 3 h and 6 h, clusters consisted of at least 4 cells in close contact are encircled by dashed lines. Scale bar: 200 µm. (**i**,**j**) Statistical analyses compare the number and the size of cell clusters formed by wild-type and MZ*lurap1* mutant cells at 3 h and 6 h. Data were collected from 6 randomly selected view fields for each condition. Bars represent the mean value ± s.d. (*, *p* < 0.05; ***, *p* < 0.001; ****, *p* < 0.0001).

Cell–cell adhesion plays an important role in radial intercalation of the DEL during epiboly [7]. To examine possible changes in the adhesive behaviors of MZ*lurap1* mutant cells, we first assayed their ability in aggregation using the hanging drop assay [22]. FLDx- or RLDx-labeled wild-type and MZ*lurap1* mutant embryos were dissociated at 30% epiboly, and equal numbers of differentially labeled cells were thoroughly mixed and co-cultured for 12 h. Confocal microscopic analysis indicated that FLDx- and RLDx-labeled wild-type cells became well intermingled, forming a seemingly compact aggregate (Figure 5a). Differentially labeled MZ*lurap1* mutant cells also became well mixed without apparent segregation, however, the resulting aggregates were not as compact as observed with wild-type cells (Figure 5b), indicating that they are less cohesive than wild-type cells. As could be expected from cell labeling and transplantation experiments (see Figure 3c–e), when differentially labeled wild-type cells and MZ*lurap1* mutant cells were cultured together, they tended to segregate from each other but preferentially aggregated according their genotypes (Figure 5c,d). This result reveals a defective adhesive property of blastoderm cells in MZ*lurap1* mutant embryos.

We further examined the effect of Lurap1 loss-of-function on cellular cohesion in a quantitative manner. Blastoderm cells were thoroughly dissociated from RLDx-labeled embryos at the four-sphere stage, and equal numbers (2 × 10^4^) of wild-type cells or MZ*lurap1* mutant cells were seeded on a fibronectin-coated substrate. Since wild-type cells tend to rapidly form small aggregates, which may interfere with statistical analyses, the presence of small clusters formed by at least four cells in close contact was counted after 3 h and 6 h of culture when intact wild-type embryos reached 70% and 100% epiboly, respectively. When randomly selected views from different experiments were imaged and analyzed by both the experimenter and an independent researcher, we found that MZ*lurap1* mutant cells displayed a strongly reduced ability in cluster formation than wild-type cells. At 3 h, wild-type cells formed an average of 28 clusters per imaged area, whereas MZ*lurap1* mutant cells formed 12 clusters (Figure 5e,f,i). At 6 h, when wild-type cells formed 36 clusters, there were 20 clusters formed by MZ*lurap1* mutant cells (Figure 5g–i). In addition, MZ*lurap1* mutant cells tended to form smaller clusters, as reflected by the average area that these clusters occupied on the culture plate (Figure 5j). Thus, the result from this quantitative analysis further confirms the observation from the aggregation assay. It suggests that loss of Lurap1 affects the adhesive affinity and leads to a reduced cohesion of blastoderm cells.

### 3.4. Disrupted Cortical F-actin Organization in the Blastoderm of MZlurap1 Mutants

**Figure 6:**
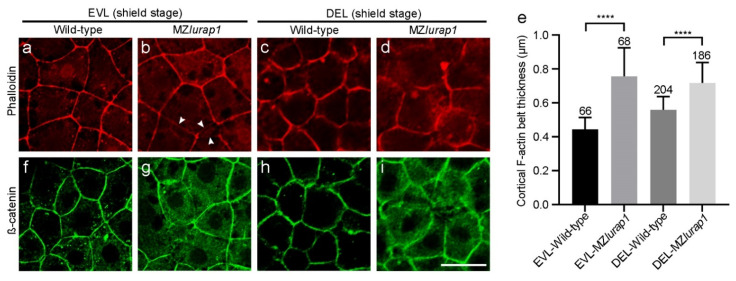
Disrupted cortical F-actin organization in the blastoderm of MZ*lurap1* mutant embryos. (**a**) Thin cortical F-actin belt is tightly organized in EVL cells of wild-type embryos. (**b**) Slightly diffuse and disrupted (arrows) cortical F-actin belt in EVL cells of MZ*lurap1* mutant embryos. (**c**) Loosely organized cortical F-actin in DEL cells of wild-type embryos. (**d**) Strongly diffuse and disrupted cortical F-actin in DEL cells of MZ*lurap1* mutant embryos. (**e**) Statistical analyses of cortical F-actin belt thickness. Data were obtained using 3 embryos from independent experiments in each condition. Numbers on the top of each column represent total cells examined in at least 3 embryos from different experiments. Bars represent the mean value ± s.d. (****, *p* < 0.0001). (**f**) Regular localization of cortical ß-catenin in EVL cells of wild-type embryos. (**g**) Disrupted and relatively diffuse localization of cortical ß-catenin in EVL cells of MZ*lurap1* mutant embryos. (**h**) ß-catenin localization in DEL cells of wild-type embryos. (**i**) Strongly disrupted ß-catenin and diffuse localization in DEL cells of MZ*lurap1* mutant embryos. Scale bar: 20 µm.

Because Lurap1 plays a role in actomyosin assembly during cell adhesion and migration [20], there is a possibility that the reduced cohesion of blastoderm cells in MZ*lurap1* mutant embryos may result from altered cytoskeletal organization. We thus examined the localization of cortical F-actin belt in blastoderm cells at the animal pole region by phalloidin staining. In wild-type embryos at the shield stage (6 hpf), cortical F-actin belt was organized tightly in the EVL (Figure 6a,c) but relatively loosely in the DEL (Figure 6c,e), reflecting tightly attached EVL cells and loosely packed DEL cells. In stage-matched MZ*lurap1* mutant embryos, cortical F-actin belt was differentially affected in EVL and DEL cells. Its localization became slightly diffuse and occasionally disrupted in the EVL (Figure 6b,e). However, severely altered localization of cortical F-actin could be observed in the DEL, which showed strongly interrupted and diffuse F-actin staining (Figure 6d,e). This result was further confirmed by immunofluorescence staining of ß-catenin, which is known to associate with actin in the cell cortex. When compared with wild-type embryos, similar disruptions of ß-catenin localization in the cortex of EVL and DEL cells were observed in MZ*lurap1* mutant embryos. The defective cortical localization of ß-catenin was closely associated with an increased cytoplasmic distribution (Figure 6f–i). Since cell adhesion molecules are linked to actin filaments through interaction with catenin [23,24], it is likely that their localization may be also affected in the absence of Lurap1 function. Together, these observations suggest that loss of Lurap1 affects F-actin organization in the blastoderm, which in turn should cause defective adhesive behaviors of the DEL in radial intercalation and lead to delayed epiboly progression.

## 4. Discussion

Lurap1 acts as a molecular scaffold to regulate the assembly of lamellar actomyosin bundles and membrane protrusion during migration of cultured cells [20], but its implication in morphogenetic movements remains unclear. We previously showed that it is required for convergence and extension movements during gastrulation in the zebrafish embryo through interaction with Dishevelled protein to regulate the non-canonical Wnt or planar cell polarity pathway [21]. In the present study, we found that it also plays a role in epiboly movement by modulating cellular adhesion through actin cytoskeleton. Loss of maternal Lurap1 affects the organization of cortical F-actin in the blastoderm, which subsequently should perturb the appropriate localization of cell adhesion molecules and cause defective cohesion of blastoderm cells, thereby preventing efficient and stable radial cell intercalation of the DEL during epiboly. These results thus identify a mechanism underlying Lurap1-mediated cell adhesion in the process of epithelial morphogenesis during early development.

Epiboly of the DEL is most severely affected in MZ*lurap1* mutant embryos in comparison with stage-matched wild-type embryos, with its vegetal movement obviously lagged further behind the EVL during gastrulation. This suggests that Lurap1 function is required for coordinated movements of the two cell layers. It was thought that changes in the adhesive behaviors of blastoderm cells, mediated in particular by E-cadherin, play an important role in the expansion of the DEL. Thus, mutation of E-cadherin gene impairs DEL cell rearrangements and delays epiboly progression [8,9,10]. We present several lines of evidence that Lurap1-mediated cell adhesion also functions to regulate radial intercalation of DEL cells. First, cell labeling of a group of DEL cells in the animal pole region of the blastula shows that they do not efficiently spread in MZ*lurap1* mutant embryos, which is associated with defective cellular protrusive behaviors. Second, cell aggregation assay indicates a reduced adhesiveness in DEL cells following loss of Lurap1 function, resulting in decreased blastoderm cohesion. Third, radial intercalation of DEL cells is not stable in MZ*lurap1* mutant embryos, largely due to balanced bi-directional cell movements that occur in the same plane. Together, these defects should collectively contribute to delayed spreading of the DEL toward the vegetal direction. Moreover, these disrupted cellular behaviors are associated with defective organization of cortical F-actin, suggesting that Lurap1 plays a role in cell adhesion during epiboly at least in part through regulation of actin cytoskeleton. Because F-actin stabilization has been shown to regulate E-cadherin assembly at adherens junctions [25,26], it is possible that the defective F-actin organization following loss of Lurap1 may secondarily affect E-cadherin subcellular localization, which in turn perturbs cell adhesion and causes unstable radial intercalation between more deep cells and more superficial cells of the DEL. Therefore, the expansion and spreading of the blastoderm over the yolk cell are impaired or delayed during gastrulation.

Cell sorting and cell adhesion experiments point out a requirement of Lurap1-mediated blastoderm cohesion in epiboly movement of the DEL. These observations suggest that cortical actin organization plays an important role in maintaining blastoderm integrity during epiboly progression. It is also consistent with previous studies showing that disruption of cell adhesion or cytoskeletal organization in the zebrafish embryo predominantly affects epiboly of DEL cells. For example, altered Gα12/13 signaling inhibits E-cadherin activity and cell adhesion, causing delayed DEL epiboly and abnormal migration of dorsal forerunner cells [16]; inhibition of cofilin 1 function perturbs actin turnover and cell adhesion between the EVL and the DEL, thus interfering with epiboly of DEL cells [27]; loss of membrane protrusions and disrupted F-actin after knockdown of angiomotin-like 2 also impair cell migration and cause epiboly delay [28]. Together, our findings demonstrate that proper actin organization is essential for cell adhesion and coordinated movements between the EVL and the DEL during epibloy.

Compared to DEL, cortical F-actin organization is mildly affected in the EVL of MZ*lurap1* mutant embryos. Although this does not seem to delay epiboly of the EVL, it may perturb the interaction of the DEL with the basal surface of EVL cells. Consistently, the interaction between DEL and EVL cells is defective in E-cadherin mutants, resulting in DEL epiboly delay [8]. Therefore, the spreading of DEL cells is closely linked to their adhesion with EVL cells, although they do not intercalate into the EVL. Indeed, it has been shown that dynamic cellular protrusions at the basal surface of EVL cells, which are dependent on F-actin content, are involved in promoting DEL rearrangements [11]. Thus, there may be a reduced protrusive activity in the EVL of MZ*lurap1* mutant embryos, which may decrease the interaction between the DEL and the EVL. As a result, radial intercalation of DEL cells becomes unstable and the expansion of the DEL is less efficient during epiboly progression.

The implication of Lurap1 in regulating cell cohesion may also influence the migratory behaviors of blastoderm cells during gastrulation. After ingression, prechordal mesendoderm cells undergo collective migration using the inner surface of the overlying epiblast as a substrate, which contributes to the elongation of anteroposterior axis [29]. It was shown that high levels of E-cadherin in prechordal mesendoderm cells are required for efficient collective migration [30]. There is also evidence that dorsal convergence of hypoblast cells and anterior migration of mesendoderm cells in zebrafish are dependent on cell–cell adhesions, cell-matrix interactions, and actin-based protrusive activities. Disruption of these processes impairs directed cell migration during gastrulation [31,32,33]. As observed in our previous work and in the present study, the migration of prechordal mesendoderm cells toward the animal pole is delayed and the elongation of anteroposterior axis is reduced in MZ*lurap1* mutant embryos. This raises the possibility that Lurap1-regulated organization of actin cytoskeleton may be involved in maintaining the cohesion of prechordal mesendoderm cells during their collective migration toward the animal pole. Thus, the activity of Lurap1 in cytoskeletal organization and adhesive interaction could exert a broad effect on cell adhesion and migration in distinct developmental processes.

Lurap1 is an adaptor protein that does not directly bind to actin, but it interacts with and activates MRCK through its leucine-rich repeats, which in turn regulates the activity of MYO18A, a PDZ domain-containing unconventional myosin [20]. In addition, Lurap1 binds to the PDZ domain of MYO18A through its PDZ-binding motif and bridges MRCK to the vicinity of MYO2A for regulating the assembly of lamellar actomyosin bundles and cell migration [20]. Thus, Lurap1 functions as an important scaffold protein and is involved in a chain of events that establish cytoskeleton dynamics to regulate cell adhesion and cell shape changes during morphogenetic movements. Further supporting a role of Lurap1-mediated adhesion in cellular behavior changes, we have previously shown that Lurap1 functionally interacts with MYO18A and p190RhoGEF, an important regulator of the Rho family of small GTPases and involved in cell motility downstream of integrins [34], in the organization of actin networks during muscle development; knockdown of *lurap1* disrupts the integrity of myofibers and reduces the ability of myoblasts to fuse with each other into multinucleated myotubes [35,36]. These observations further support an implication of Lurap1 in connecting actin cytoskeleton to cell adhesion. Given the importance of MYO18A and p190RhoGEF in the regulation of the actin-based contractile machinery in different cell types [37,38,39], it would be of interest to determine whether these Lurap1 interaction partners are also involved in epiboly movement.

## 5. Conclusions

Our results identify Lurap1 as a regulator of cell behavior changes required for orchestrating coordinated cell movements during epithelial morphogenesis. Specifically, Lurap1 plays a role in cellular cohesion of the blastoderm through regulation of cortical cytoskeletal organization to maintain blastoderm cohesion and stabilize radial cell intercalation, thus contributing to epiboly progression. This work reveals a novel role of Lurap1 in the earliest morphogenetic process of gastrulation. It provides insights on the mechanism underlying the differential adhesion of blastoderm cells during epiboly movement.

## Data Availability

Data supporting reported results can be found in the manuscript, and information on the *lurap1* mutant line can be found at www.zfin.org (accessed on 15 December 2021).

## Supplementary Materials

The following are available online at https://www.mdpi.com/article/10.3390/biology10121337/s1, Video S1: Movements of DEL cells in a wild-type embryo, Video S2: Movements of DEL cells in an MZ*lurap1* mutant embryo.

## Appendix A

A time-lapse recording performed at 50% epiboly shows predominant outward movements of more deep cells (blue) and limited inward movements of more superficial cells (red) in a wild-type embryo (Video S1).

## Appendix B

Time-lapse recording performed at 50% epiboly shows balanced bi-directional movements between more deep cells (blue) and more superficial cells (red) in an MZ*lurap1* mutant embryo (Video S2).

## References

[1] Solnica-Krezel L. (2005). Conserved patterns of cell movements during vertebrate gastrulation. Curr. Biol..

[2] Kimmel C.B., Ballard W.W., Kimmel S.R., Ullmann B., Schilling T.F. (1995). Stages of embryonic development of the zebrafish. Dev. Dyn..

[3] Warga R.M., Kimmel C.B. (1990). Cell movements during epiboly and gastrulation in zebrafish. Development.

[4] Bruce A.E.E. (2016). Zebrafish epiboly: Spreading thin over the yolk. Dev. Dyn..

[5] Bensch R., Song S., Ronneberger O., Driever W. (2013). Non-directional radial intercalation dominates deep cell behavior during zebrafish epiboly. Biol. Open.

[6] Bruce A.E.E., Heisenberg C.P. (2020). Mechanisms of zebrafish epiboly: A current view. Curr. Top. Dev. Biol..

[7] Solnica-Krezel L. (2006). Gastrulation in zebrafish—All just about adhesion?. Curr. Opin. Genet. Dev..

[8] Kane D.A., McFarland K.N., Warga R.M. (2005). Mutations in half baked/E-cadherin block cell behaviors that are necessary for teleost epiboly. Development.

[9] McFarland K.N., Warga R.M., Kane D.A. (2005). Genetic locus half baked is necessary for morphogenesis of the ectoderm. Dev. Dyn..

[10] Shimizu T., Yabe T., Muraoka O., Yonemura S., Aramaki S., Hatta K., Bae Y.K., Nojima H., Hibi M. (2005). E-cadherin is required for gastrulation cell movements in zebrafish. Mech. Dev..

[11] Rutherford N.E., Wong A.H., Bruce A.E.E. (2019). Spatiotemporal characterization of dynamic epithelial filopodia during zebrafish epiboly. Dev. Dyn..

[12] Lee S.J. (2014). Dynamic regulation of the microtubule and actin cytoskeleton in zebrafish epiboly. Biochem. Biophys. Res. Commun..

[13] Cheng J.C., Miller A.L., Webb S.E. (2004). Organization and function of microfilaments during late epiboly in zebrafish embryos. Dev. Dyn..

[14] Köppen M., Fernandez B.G., Carvalho L., Jacinto A., Heisenberg C.P. (2006). Coordinated cell-shape changes control epithelial movement in zebrafish and Drosophila. Development.

[15] Lachnit M., Kur E., Driever W. (2008). Alterations of the cytoskeleton in all three embryonic lineages contribute to the epiboly defect of Pou5f1/Oct4 deficient MZspg zebrafish embryos. Dev. Biol..

[16] Lin F., Chen S., Sepich D.S., Panizzi J.R., Clendenon S.G., Marrs J.A., Hamm H.E., Solnica-Krezel L. (2009). Galpha12/13 regulate epiboly by inhibiting E-cadherin activity and modulating the actin cytoskeleton. J. Cell Biol..

[17] Sun Q., Liu X., Gong B., Wu D., Meng A., Jia S. (2017). Alkbh4 and atrn act maternally to regulate zebrafish epiboly. Int. J. Biol. Sci..

[18] Li Y.L., Shao M., Shi D.L. (2017). Rac1 signalling coordinates epiboly movement by differential regulation of actin cytoskeleton in zebrafish. Biochem. Biophys. Res. Commun..

[19] Li Y.L., Cheng X.N., Lu T., Shao M., Shi D.L. (2021). Syne2b/nesprin-2 is required for actin organization and epithelial integrity during epiboly movement in zebrafish. Front. Cell Dev. Biol..

[20] Tan I., Yong J., Dong J.M., Lim L., Leung T. (2008). A tripartite complex containing MRCK modulates lamellar actomyosin retrograde flow. Cell.

[21] Cheng X.N., Shao M., Li J.T., Wang Y.F., Qi J., Xu Z.G., Shi D.L. (2017). Leucine repeat adaptor protein 1 interacts with Dishevelled to regulate gastrulation cell movements in zebrafish. Nat. Commun..

[22] Carreira-Barbosa F., Kajita M., Morel V., Wada H., Okamoto H., Martinez Arias A., Fujita Y., Wilson S.W., Tada M. (2009). Flamingo regulates epiboly and convergence/extension movements through cell cohesive and signalling functions during zebrafish gastrulation. Development.

[23] Schepis A., Sepich D., Nelson W.J. (2012). αE-catenin regulates cell-cell adhesion and membrane blebbing during zebrafish epiboly. Development.

[24] Yap A.S., Crampton M.S., Hardin J. (2007). Making and breaking contacts: The cellular biology of cadherin regulation. Curr. Opin. Cell Biol..

[25] Hong S., Troyanovsky R.B., Troyanovsky S.M. (2013). Binding to F-actin guides cadherin cluster assembly, stability, and movement. J. Cell Biol..

[26] Wu S.K., Gomez G.A., Michael M., Verma S., Cox H.L., Lefevre J.G., Parton R.G., Hamilton N.A., Neufeld Z., Yap A.S. (2014). Cortical F-actin stabilization generates apical-lateral patterns of junctional contractility that integrate cells into epithelia. Nat. Cell Biol..

[27] Lin C.W., Yen S.T., Chang H.T., Chen S.J., Lai S.L., Liu Y.C., Chan T.H., Liao W.L., Lee S.J. (2010). Loss of cofilin 1 disturbs actin dynamics, adhesion between enveloping and deep cell layers and cell movements during gastrulation in zebrafish. PLoS ONE.

[28] Huang H., Lu F.I., Jia S., Meng S., Cao Y., Wang Y., Ma W., Yin K., Wen Z., Peng J. (2007). Amotl2 is essential for cell movements in zebrafish embryo and regulates c-Src translocation. Development.

[29] Tada M., Kai M. (2012). Planar cell polarity in coordinated and directed movements. Curr. Top. Dev. Biol..

[30] Montero J.A., Carvalho L., Wilsch-Bräuninger M., Kilian B., Mustafa C., Heisenberg C.P. (2005). Shield formation at the onset of zebrafish gastrulation. Development.

[31] Nair S., Schilling T.F. (2008). Chemokine signaling controls endodermal migration during zebrafish gastrulation. Science.

[32] Love A.M., Prince D.J., Jessen J.R. (2018). Vangl2-dependent regulation of membrane protrusions and directed migration requires a fibronectin extracellular matrix. Development.

[33] Prince D.J., Jessen J.R. (2019). Dorsal convergence of gastrula cells requires Vangl2 and an adhesion protein-dependent change in protrusive activity. Development.

[34] Miller N.L., Lawson C., Chen X.L., Lim S.T., Schlaepfer D.D. (2012). Rgnef (p190RhoGEF) knockout inhibits RhoA activity, focal adhesion establishment, and cell motility downstream of integrins. PLoS ONE.

[35] Cao J.M., Cheng X.N., Li S.Q., Heller S., Xu Z.G., Shi D.L. (2016). Identification of novel MYO18A interaction partners required for myoblast adhesion and muscle integrity. Sci. Rep..

[36] Cao J.M., Li S.Q., Shao M., Cheng X.N., Xu Z.G., Shi D.L. (2014). The PDZ-containing unconventional myosin XVIIIA regulates embryonic muscle integrity in zebrafish. J. Genet. Genom..

[37] Taft M.H., Latham S.L. (2020). Myosin XVIII. Adv. Exp. Med. Biol..

[38] Ouyang Z., Zhao S., Yao S., Wang J., Cui Y., Wei K., Jiu Y. (2021). Multifaceted function of myosin-18, an unconventional class of the myosin superfamily. Front. Cell Dev. Biol..

[39] Miller N.L., Kleinschmidt E.G., Schlaepfer D.D. (2014). RhoGEFs in cell motility: Novel links between Rgnef and focal adhesion kinase. Curr. Mol. Med..

